# Development and validation of the Remote Working Benefits & Disadvantages scale

**DOI:** 10.1007/s11135-022-01364-2

**Published:** 2022-04-29

**Authors:** Emanuela Ingusci, Fulvio Signore, Claudio Giovanni Cortese, Monica Molino, Paola Pasca, Enrico Ciavolino

**Affiliations:** 1 Department of History, Society and Human Studies, University of Salento, Studium 2000 - Building 5 - Via di Valesio, 24 - Lecce (LE) Office 18, Ground Floor, Lecce, Italy; 2 Department of Psychology, University of Turin, Via Verdi 10, 10124 Torino, Italy; 3 Computer Science and New Technology, WSB University, 80-266 Gdańsk, Poland

**Keywords:** Remote-working, SEM, Scale, Validation, Benefits, Disadvantages

## Abstract

The changes that are constantly occurring in the labour sector have led organisations and companies to move towards digital transformation. This process was accelerated by the COVID-19 pandemic and  conducted to a massive recourse to the practice of remote working, which in this study is understood as the term for the way of performing work outside the usual workplace and with the support of ICT. Currently, there are no flexible scales in the literature that allow measuring the benefits and disadvantages of remote working with a single instrument. Thus, the distinction between the positive and negative consequences of working remotely, substantiated by a solid literature, provides a framework for a systematical understanding of the issue. The aim of the present study is to develop and validate a scale on remote working benefits and disadvantages (RW-B&D scale). For this end, a preliminary Exploratory Factor Analysis (EFA) with 304 participants, a tailored EFA with a sample of 301 workers and a Confirmatory Factor Analysis (CFA) with 677 workers were conducted. Participants were all Italian employees who worked remotely during the period of the COVID-19 health emergency. Data were collected between October 2020 and April 2021. The psychometric robustness of the model was assessed through bootstrap validation (5000 resamples), fit indices testing and measurement of factorial invariance. The statistical analyses demonstrated the bifactorial nature of the scale, supporting the research hypothesis. The model showed good fit indices, bootstrap validation reported statistically significant saturations, good reliability indices, and convergent and discriminant validity. Measurement invariance was tested for gender and organisational sector. The results suggested that the novel scale facilitates the quantitative measurement of the benefits and disadvantages associated with remote working in empirical terms. For this reason, it could be a streamlined and psychometrically valid instrument to identify the potential difficulties arising from remote working and, at the same time, the positive aspects that can be implemented to improve organisational well-being.

## Introduction

Over the last decades, companies and institutions have become increasingly aware of the potential of using technology in work and everyday life: Facilitating processes and speeding up operations can improve working conditions and job demands (Polák 2017). Although there is evidence that digital technology is triggering fundamental social change and economic growth, there is wide variation in its integration across European countries, with Scandinavian and other small countries at the top and Bulgaria, Romania, and Greece at the bottom (European Commission 2019a, b). Against this backdrop, the pandemic had a transformative effect: first, it led to an exponential acceleration of technology use, significantly changing the perception of digitalization in our economies and societies; second, it contributed to the spread of technology use in our daily lives and to organizations becoming more environmentally aware and sustainable. Most of the companies surveyed by the European Commission state that Information and Communication Technology (hereafter ICT) has helped to reduce the number of business trips, the consumption of materials, equipment, and consumables, and waste production, and, above all, has made it easier to work in remote locations (European Commission 2021). The latter is particularly important not only from the point of view of the company, but also from the point of view of the employees, as it has enabled them to continue working in complete safety and to ensure continuity and support in the event of a health emergency.

In the literature, different terms are used for flexible work arrangements, for example, remote working, teleworking or home working (Savić 2020; Vartiainen 2021). In general, they refer to those employees who work from home, specifically: the employee is bound to the company by an employment relationship; he/she is formally obliged to perform certain work tasks; he/she works outside the physical space of the company, but can telecommunicate with the employer and colleagues. In line with the methodological principle of parsimony, this paper focuses on the common thread connecting the different virtual work typologies - i.e., working from a location other than the traditional office and using technology to complete work-related tasks as identified by Allen et al. (2015) - rather than delving further into the definitions of each term (Hübener 1983). Regardless of the definitions, all authors agree on what remote work entails, i.e., its benefits and disadvantages.

From a theoretical perspective, three main streams can be identified in the literature. Two of them consider remote working as an independent variable that affects work outcomes; in these approaches, work characteristics are studied either as a mediator (Vander Elst et al. 2017) or as a moderator (Golden and Gajendran 2019). Both approaches, however, consider an individual’s work as a whole that includes both remote and non-remote components, with the independent variable capturing the extent of each aspect. A third approach focuses only on the experience of remote working. Researchers in this area are interested in understanding how the characteristics of virtual work shape the work experience in the context of the home work environment (Bentley et al. 2016; Kossek et al. 2009). When the COVID-19 pandemic made remote working the “new normal", the latter approach became particularly relevant, although few studies have considered it. For example, Wang et al. (2021) examined the main challenges faced by remote workers in the context of the pandemic and the role of virtual work characteristics in shaping these challenges. They identified four challenges (work-home interference, ineffective communication, procrastination, and loneliness) and four characteristics (social support, job autonomy, monitoring, and workload) that may influence perceptions of these challenges. The study showed that the benefits of working from home play a protective role against work challenges, loneliness, and procrastination tendencies. More recently, Slavković et al. (2022) emphasized the importance of social support as a moderator/mediator between organizational antecedents and consequences. From a measurement standpoint, the advantages and disadvantages of remote working have been considered as independent monads: in some cases, by adopting constructs that originally belonged to other scales, as in the case of the social support items of the Work Design Questionnaire (Morgeson and Humphrey 2006); in other cases, by creating ad hoc items, as in the case of assessing the degree of supervision of employees during virtual work (Wang et al. 2021); and finally, by adapting scales developed in other contexts to the domestic work context, as in the case of the time-based interference constructs of Carlson’s Work-Family Conflict scale (Carlson et al. 2000) or the Tuckman’s Procrastination scale (Tuckman 1991).

Table 1 provides an overview of recent studies that examined remote working during the pandemic. The measures of remote working included 19, 29, 48, 37 items and 17 items, respectively. A first detail is that none of the studies examined the relationship with technology and how it changed. Second, there is little consistency in capturing relevant aspects of remote working across the studies: the discrepancy in the items and constructs covered highlights a likely tendency to emphasise some aspects of virtual work to a greater or lesser extent, while neglecting others that might be relevant. In addition, the use of nonspecific scales for the phenomenon makes its measurement inconsistent and time consuming. However, among the first efforts to systematize a measure focused on analysing the well-being-related topics for e-workers is that of Grant et al. (2019). Indeed, this scale offers three levels of assessment: individual, supervisory and organisational, allowing for the capture of characteristics related to the worker’s psychological well-being. However, this scale cannot specifically gauge the advantages and disadvantages of remote working, as it contains several dimensions that can essentially relate to positive and negative consequences of remote working. For instance, the literature suggests a new way to systematize the phenomenon: Harpaz (2002) distinguishes between advantages and disadvantages of remote working at the individual, organizational, and societal levels. Later studies that either offer a synthesis of the phenomenon (Martin and MacDonnell 2012) or explore it further (Tremblay and Thomsin 2012; Savić 2020) also conceptualize it in terms of benefits and disadvantages, although they include different constructs. It seems that conceptualizing remote working in terms of its advantages and disadvantages is a unifying, comprehensive way to define and operationalize it.

**Table 1:** Overview of recent studies examining remote working and its dimensions during the pandemic

Author	Construct	Source of the scale	No. Items
Slavković et al. (2022)	work engagement	Schaufeli et al. (2006)	4
	job performance	Williams and Anderson (1991)	3
	work-home/home-work interaction	Geurts et al. (2005)	6
	loneliness	Russell et al. (1980)	3
	social support	Morgeson and Humphrey (2006)	3
Wang et al. (2021)	social support	Morgeson and Humphrey (2006)	4
	job autonomy	Hackman and Oldham (1980)	3
	received monitoring	built ad-hoc	4
	self-discipline	Lindner et al. (2015)	3
	work-home/home-work interference	Carlson et al. (2000)	6
	procrastination	Tuckman (1991)	3
	loneliness	Russell et al. (1980)	3
	communication effectiveness	Lowry et al. (2009)	3
Angelici and Profeta (2020)	productivity	ad-hoc built	10
	flexibility		4
	well-being		13
	work-life balance		17
	commitment		4
Prasad et al. (2020)	team work		4
	communication		5
	peer		3
	job related factors	Prasad et al. (2016)	5
	organization policies	Prasad et al. (2018)	6
	organization climate	Prasad and Vaidya (2018)	5
	job satisfaction		4
	psychological factors		5
Grant et al. (2019)	work-life interference	ad-hoc built	7
	effectiveness/productivity		4
	organizational trust		3
	flexibility		3

### Objectives and articulation of the paper

In accordance with a review of the existing literature on the issue, it is clear that a unified, specific, and comprehensive measurement tool for the phenomenon is still lacking. This paper aims to fill the gap of detecting an unified and consistent instrument that provides researchers with a reference point for measuring the recent widespread phenomenon of remote working by developing a measurement scale, the Remote-Working Benefits & Disadvantages scale (hereafter RW-B&D scale), which unifies the results of the literature review. Specifically, the research purposes relevant to the development of the tool are:

- *Aim 1*: to explore the factorial structure *RW-B&D* scale;
- *Aim 2*: to investigate the psychometric properties of the scale (i.e., internal consistency, reliability, convergent and divergent validity);
- *Aim 3*: to evaluate measurement invariance as a function of gender and sector.

To test the first goal ($Aim_{1}$), two Exploratory Factor Analysis (EFA) were conducted, while the other two targets ($Aim_{2}$ and $Aim_{3}$) were investigated by Confirmatory Factor Analysis (CFA) and the measurement invariance procedure, respectively (Bollen 1989; Costello and Osborne 2005; Ciavolino et al. 2015). In the introduction we offered a description of remote working and its relevance, a general foreword on the potential advantages and disadvantages of remote working in general and and a detailed comparative review of the main measurement tools available and their critical issues. The introduction is followed by the general objectives and the outline of the paper (sect. 1.1). In the second section we described the scale development, by highlighting the needs identified in the literature, the scale development procedure, an examination of the benefits and disadvantages of remote working and a discussion of the bifactorial nature of the measurement scale. The pilot study (Sect. 3), study 1 (Sect. 4) and study 2 (Sect. 5) are then presented with their respective sample and data description, methodology, and results. Discussion and conclusions are addressed in Sect. 6, while limitations and practical implications are discussed in Sect. 7.

## Scale development

Thus, a review of the existing literature highlights the need for a unified and consistent instrument that provides researchers with a reference point for measuring the recent widespread phenomenon of remote working. The RW-B&D scale, developed following the methodological guidelines of Boateng et al. (2018), is the result of this effort.

### Procedure

Taking into account the careful review of the literature and the critical issues highlighted therein, the unifying notion of remote working in terms of advantages and disadvantages, and considering further feedback from privileged witnesses, a pool of 33 items was created to measure the advantages and disadvantages of remote working, based on the integration of top-down (literature review) and bottom-up (focus group) classification methods, as suggested by the guidelines by Boateng et al. (2018).

First, a pilot study, involving 304 subjects, confirmed a two-dimensional factorial structure as the best solution for the scale by testing an EFA. However, to provide a transversal and generalizable instrument, some items (explicitly related to the pandemic or to the presence of children due to the closure of schools) were deleted. In addition, the analyses showed a strong collinearity between some items: this aspect allowed, on the one hand, to identify redundant items and, on the other hand, to combine and reformulate items of the same polarity. Finally, we arrived at a version of the scale with 14 items. In study 1, the factorial structure of the 14-item scale and its statistical robustness were tested with a sample of 301 participants. Subsequently, a second EFA was conducted to verify whether the assumed bifactorial structure was still reflected in the data. These two studies, based on a total of 605 individuals, formed the basis for conducting the confirmatory study.

Finally, study 2 was conducted on 677 workers for confirmatory purposes. In addition to assessing factorial goodness of fit with appropriate fit indices, important psychometric properties such as reliability, validity, and measurement invariance were also tested. The decision to split the sample into two subsamples with approximately the same number of subjects was based on the recommendations of Hair et al. (2019), according to which exploratory and confirmatory analyses are best conducted when the total sample is evenly (and randomly) distributed based on available observations.

#### The main advantages of remote working

Literature on remote working pointed out that there are some connected advantages and disadvantages. According to Savić (2020) and Prasad et al. (2020), in addition to cost savings, the benefits of virtual working include greater job satisfaction, higher productivity and flexibility, reduced demands, better work-life balance, and higher employee retention, especially among older generations; moreover, environmental benefits have been highlighted (European Commission 2021). Regarding the physical context, distance from the workplace requires some self-discipline on the part of the employee to maintain the pace of work. Angelici and Profeta (2020) conducted a randomised controlled trial comparing remote working (referred to by the authors as ’smart working’) with traditional on-site work. They concluded that ’smart working’ has mostly positive effects, especially for women: in addition to higher productivity, greater satisfaction with one’s salary, and greater well-being, the perception of spending more time on housework and family care activities is observed. The authors suggest that these effects are due to a different work organisation characterised by more efficient time management (Angelici and Profeta 2020).

#### The main disadvantages of remote working

In contrast, the disadvantages relate to the sociological and psychological challenges posed by isolation and by the “autonomy paradox", where greater autonomy and flexibility leads to a more erratic pace of work, either due to personal ambition or remote control by the organisation. All these aspects may ultimately lead to a change in working hours and an increase in work-life conflict (Eurofound 2020), also due to the lack of separation between work and private life. Finally, privacy and security issues are also critical (Savić 2020). The literature on the subject also suggests that some of the practices involved in working remotely serve a dual role: on the one hand they offer a favourable effect, but at the same time they might represent a crucial element: although physical distance may bring about a perception of greater autonomy, on the other hand it could provoke a lack of commitment and identification with the values and culture of the company, as well as a natural and consequent sense of isolation (Molino et al. 2020; Angelici and Profeta 2020).

### The focus group

Based on the unifying vision of remote working in terms of benefits and disadvantages, several steps were taken to create a draft scale. First, the literature was studied in depth to identify the constructs associated with remote working and the methods used to measure them. In addition, a focus group with privileged witnesses (i.e., public or private sector workers who have benefited from remote working) allowed for both identification of aspects not addressed in the literature and verification that existing items represented workers’ concerns. The focus group participants were individuals who were selected “by reasoned choice", according to the objectives of the survey. They had similar characteristics and practiced their profession in similar contexts. In particular, the sample was made up of 12 Italian employees, equally distributed between men and women, in both the public and private sectors. Research was conducted through focus group discussion (FGD), as mentioned above. Focus group is a qualitative data collection technique used in social research based on the information that emerges from a collective discussion about a topic or a subject that the researcher wishes to investigate (Wilkinson 2004). Thus, the objective is to thoroughly explore the opinions, attitudes and motivations of the stakeholders involved in the psychological phenomenon at hand(Wilkinson 2004).

### The results of focus group and the process of formulating the items

The focus group discussion also contributed to thinking about how the items could be generic (not specific to the pandemic emergency) but still applicable to a wide range of workers. In relation to the dimensions that participants identified as important aspects in defining what it means to be a remote worker, they mentioned feeling less protected (e.g., in terms of work and recovery rhythms), having greater difficulty receiving recognition for their work, and consequently advancing in their careers.

Thus, based on the latter suggestions and the existing literature analysis, in order to create a first draft of the questionnaire, a number of integrations and modifications were made. Specifically, concepts already present in the literature were reduced and summarised in terms of benefits - i.e., autonomy and flexibility, self-discipline, work-life balance, social support, perceived productivity, job satisfaction, and well-being - and disadvantages - loneliness and isolation, perceived supervision by supervisors, work-related factors such as reduced visibility, limited access to training and information, home-work interference. Concepts for which there are no formal measurement tools, as well as those arising from privileged witnesses - pragmatic benefits such as savings in travel time and money, improved use of technology, or disadvantages such as difficult access to career advancement or protection - were created on an ad hoc basis. Specifically, item development followed the categorization between the two dimensions identified in the literature, i.e., benefits and disadvantages.

The positive aspects of remote working that were considered in the formulation of the items included: the positive impact on work-life balance (Bentley et al. 2016; Felstead and Henseke 2017), economic savings mainly (but not only) due to reduced travel (Slavković et al. 2022), stress reduction (Klopotek 2017), better organisation, planning, and autonomy of one’s work (Wang et al. 2021), which also has a positive impact on relationships with colleagues (Morgeson and Humphrey 2006), and better use of available technology (Phillips 2020). These elements lead to improved satisfaction with one’s work, especially after an initial period when the experience of remote working can be traumatic (Prasad et al. 2020).

On the contrary, the other side of the coin leads to think about the negative and often hidden consequences of this new way of working. Among the most critical aspects are the lack of sociability and identification with one’s own organisation and workplace (Toscano and Zappalà 2020a, b), as well as the purely logistical aspects resulting from the physical impossibility of going to the office to access necessary tools or information (Gheno and Pesenti 2021; Bonacini et al. 2021). Unfavourable aspects also include perceptions of lower visibility or recognition of one’s work (Toscano and Zappalà 2020b) and lower perceptions of career and protection advancement opportunities (Mulki et al. 2009; Arntz et al. 2020). Finally, areas considered highly problematic include feeling constantly monitored (Leonardi et al. 2010; Bondanini et al. 2020), receiving negative comments from colleagues or supervisors (Mulki et al. 2009), and difficulty concentrating or distracting oneself from work because of the intrusive nature of new technologies (Molino et al. 2020; Bondanini et al. 2020). Therefore, the original questionnaire, which would later be subjected to psychometric analysis, consisted of 33 items.

## Pilot study

The initial analyses were therefore conducted on a sample of 304 Italian workers who completed an online questionnaire. Recruitment was done through a snowball convenience sampling procedure. Participants were assured anonymity and the characteristics of the survey were explained. Participation was voluntary and data were analysed in aggregate form. Frequency analysis was performed (Corallo et al. 2020). From a purely sociodemographic perspective, participants were fairly balanced in terms of gender (52.1% women and 47.9% men). About half of the subjects lived with a partner and children (58.6%), had a university degree (45.4%), and were still working remotely at the time of the survey (53.4%). The survey period was from October 2020 to April 2021, and although all participants had to work remotely due to the pandemic emergency, some were working in person at the time of data collection. The mean age of the sample was 51.5 years, with a standard deviation of 7.2.

### Results

According to the criterion of eigenvalues greater than 1, the first EFA results suggested the choice of 3 dimensions, which cumulatively explained 44.4% of the variance. However, the third dimension not only had an eigenvalue only slightly greater than 1, but also explained only an additional 4% of the variability in the data. In addition, it included contradictory items. These statistical considerations suggested that the bifactorial solution (which accounted for the 40.4% of the information) was more appropriate. Another problem concerned a strong collinearity between some items (see Table 2 and Table 3), which for this reason were appropriately reformulated by integrating different aspects into the same theme.

**Table 2:** Correlation matrix of pilot study’s benefits’ items.

	BEN1	BEN2	BEN3	BEN4	BEN5	BEN6	BEN7	BEN8	BEN9	BEN10	BEN11	BEN12	BEN13	BEN14	BEN15
BEN1	–														
BEN2	0.360***	–													
BEN3	0.399***	0.680***	–												
BEN4	0.288***	0.376***	1.000***	–											
BEN5	0.388***	0.310***	0.349***	0.423***	–										
BEN6	0.286***	0.572***	0.537***	0.337***	0.283***	–									
BEN7	0.248***	0.537***	0.592***	0.422***	0.256***	0.577***	–								
BEN8	0.299***	0.526***	0.670***	0.404***	0.300***	0.437***	0.600***	–							
BEN9	0.249***	0.464***	0.414***	0.292***	0.272***	0.398***	0.338***	0.315***	–						
BEN10	0.106	0.306***	0.326***	0.207***	0.110	0.374***	0.239***	0.292***	0.146*	–					
BEN11	0.075	0.337***	0.351***	0.221***	0.141*	0.368***	0.257***	0.352***	0.295***	0.542***	–				
BEN12	0.353***	0.553***	0.485***	0.330***	0.354***	0.551***	0.373***	0.419***	0.471***	0.390***	0.450***	–			
BEN13	0.222***	0.482***	0.412***	0.295***	0.286***	0.468***	0.356***	0.393***	0.362***	0.455***	0.446***	0.595***	–		
BEN14	0.269***	0.483***	1.000***	1.000***	0.263***	0.578***	0.397***	0.394***	0.330***	0.536***	0.537***	0.635***	0.638***	–	
BEN15	0.160**	0.373***	0.379***	0.368***	0.297***	0.420***	0.359***	0.387***	0.392***	0.312***	0.405***	0.487***	0.463***	0.489***	–

*p < .05, **p < .01, ***p < .001

**Table 3:** Correlation matrix of pilot study’s disadvantages’ items.

	DISADV1	DISADV2	DISADV3	DISADV4	DISADV5	DISADV6	DISADV7	DISADV8	DISADV9	DISADV10	DISADV11	DISADV12	DISADV13	DISADV14	DISADV15	DISADV16	DISADV17	DISADV18
DISADV1	–																	
DISADV2	0.637***	–																
DISADV3	0.441***	0.396***	–															
DISADV4	0.450***	0.351***	0.556***	–														
DISADV5	0.416***	0.499***	0.254***	0.240***	–													
DISADV6	0.477***	0.548***	0.464***	0.457***	0.530***	–												
DISADV7	0.447***	0.398***	0.261***	0.357***	0.416***	0.412***	–											
DISADV8	0.351***	0.262***	0.333***	0.387***	0.256***	0.304***	0.392***	–										
DISADV9	0.417***	0.338***	0.411***	0.424***	0.324***	0.448***	0.321***	0.346***	–									
DISADV10	0.450***	0.373***	0.483***	0.505***	0.312***	0.464***	0.518***	0.396***	0.429***	–								
DISADV11	0.497***	0.496***	0.534***	0.534***	0.330***	0.542***	0.393***	0.246***	0.466***	0.514***	–							
DISADV12	0.234***	0.231***	0.322***	0.346***	0.124*	0.232***	0.170**	0.327***	0.288***	0.275***	0.370***	–						
DISADV13	0.391***	0.266***	0.427***	0.549***	0.191***	0.341***	0.339***	0.308***	0.333***	0.466***	0.517***	0.337***	–					
DISADV14	0.420***	0.330***	0.470***	0.492***	0.209***	0.342***	0.327***	0.298***	0.329***	0.447***	0.487***	0.297***	0.583***	–				
DISADV15	0.535***	0.576***	0.373***	0.383***	0.438***	0.533***	0.454***	0.237***	0.404***	0.365***	0.434***	0.193***	0.269***	0.310***	–			
DISADV16	0.352***	0.306***	0.446***	0.466***	0.207***	0.333***	0.309***	0.299***	0.453***	0.506***	0.380***	0.313***	0.348***	0.335***	0.340***	–		
DISADV17	0.445***	0.375***	0.229***	0.286***	0.372***	0.329***	0.483***	0.207***	0.270***	0.299***	0.294***	0.146*	0.293***	0.193***	0.310***	0.206***	–	
DISADV18	0.423***	0.448***	0.292***	0.289***	0.455***	0.380***	0.473***	0.447***	0.358***	0.358***	0.332***	0.279***	0.236***	0.253***	0.384***	0.311***	0.357***	–

*p < .05, **p < .01, ***p < .001

Furthermore, items that explicitly referred to the role of remote working in emergency confinement period were deleted, as were those that mentioned children or partners (replaced by a more general reference to personal life). All of these reasons, combined with the theoretical issues described below, resulted in the number of items in the questionnaire being reduced from 33 to 14 (7 benefits and 7 disadvantages). The final wording of the items is presented in Table 4 (English version) and Table 5 (Italian version).

The response scale included a 4-point Likert scale, where 1 = Not at all; 2 = A little; 3 = Quite a lot; 4 = Totally; N/A = Not applicable (in case there are situations that do not fit the subjects’ personal lives). The heading of the scale was: “*In your experience, the use of alternative forms of work (smart working/telecommuting) can lead to the following: ...".*

**Table 4:** English version of the RW-B&D scale’s items

N	Item	Dimension
1	Better possibility to coordinate work-family balance and/or to meet family needs in an appropriate way	Benefits
2	Economical and/or time saving in travelling	Benefits
3	Stress reduction and more time available for oneself	Benefits
4	Possibility of independently working and/or better concentration, organisation/planning of one’s work	Benefits
5	Better relationship with colleagues and/or superiors	Benefits
6	Increased job satisfaction	Benefits
7	Better use of available technology	Benefits
8	Loss of sense of belonging to one’s office, isolation and lack of socialisation with colleagues	Disadvantages
9	Reduced visibility towards superiors and/or recognition of own work	Disadvantages
10	Difficulty in accessing tools/documents in the office/office and obtaining information from colleagues who work in the office	Disadvantages
11	Difficulty in planning work and/or excessive rigidity in working time	Disadvantages
12	Less access to professional training and/or career progression, perception of less protection and/or less access to information on work decisions	Disadvantages
13	Perception of being subjected to stricter controls and/or negative perception by colleagues or superior	Disadvantages
14	Difficulty in concentrating due to domestic distractions and/or technology used	Disadvantages

**Table 5:** Items of questionnaire in Italian

N	Item	Dimension
1	Maggiori possibilità di coordinare meglio sfera privata e sfera lavorativa e/o di rispondere alle esigenze familiari in modo adeguato	Benefici
2	Risparmio economico e/o di tempo negli spostamenti	Benefici
3	Riduzione dello stress e/o piú tempo disponibile per sé stessi	Benefici
4	Possibilità di lavorare in modo autonomo e/o di migliore concentrazione, organizzazione/pianificazione del proprio lavoro	Benefici
5	Migliore rapporto con i colleghi e/o superiori	Benefici
6	Aumento della soddisfazione rispetto al proprio lavoro	Benefici
7	Migliore utilizzo della tecnologia a disposizione	Benefici
8	Perdita di senso di appartenenza al proprio ufficio, isolamento e mancanza di socializzazione con colleghi	Svantaggi
9	Riduzione della propria visibilità nei confronti dei superiori e/o del riconoscimento del proprio lavoro	Svantaggi
10	Difficoltà ad accedere a strumenti/documenti presenti in ufficio/sede ed avere informazioni rispetto a colleghi e colleghe che lavorano in sede	Svantaggi
11	Difficoltà nella pianificazione del lavoro e/o eccessiva rigidità nei tempi di lavoro	Svantaggi
12	Minore accesso alla formazione professionale e/o progressioni di carriera, percezione di minore tutela e/o di minore accesso alle informazioni sulle decisioni lavorative	Svantaggi
13	Percezione di essere sottoposto/a a controlli piú rigorosi e/o percezione negativa da parte dei colleghi o del superiore gerarchico	Svantaggi
14	Difficoltà di concentrazione a causa di distrazioni domestiche e/o delle tecnologie utilizzate	Svantaggi

## Study 1: exploratory factor analysis

For the analyses we used *Jamovi*, version 1.8 and *RStudio*, version 1.4.1717.

EFA was conducted on a sample of 301 workers who experienced a period of remote working during the COVID-19 health crisis. Participants were selected using a non-probability snowball convenience sampling method. Subjects were intercepted throughout the Italian territory between November 2020 and April 2021. They completed an online questionnaire.

In terms of demographic characteristics, 57.5% were men and 42.5% were women. The mean age was 39.7 years (SD = 12.7), ranging from 18 to 66. The most frequent age was 30 years old. 38.3% of the sample had a high school diploma, 48% had a university degree and 13.7% had a post-lauream degree. The majority of subjects had a partner (61.7%) and no dependent children (58.7%). In addition, 62.7% had a permanent contract, 21.3% a fixed-term contract, and 9% were self-employed. Most of the sample worked for a private company (62.3%), while 37.7% worked for a public organization. Finally, regarding the distance to the workplace, for 36.3% of the individuals it was between 10 and 25 kilometers, for 30.7% between 1 and 5 kilometers and for 13.7% between 25 and 50 kilometers.

### Results

Skewness and kurtosis of benefits and disadvantages of remote working were in the range of ± 1.96 (Gravetter et al. 2020) and the 14 items of the scale had excellent measures of sampling adequacy (KMO = 0.87 and Bartlett’s Test of Sphericity < .001; Kaiser , 1974). The EFA was based on two dimensions, as suggested by the development of our scale. We performed the analysis by using an oblimin rotation and we found that the 14 items fit this procedure well. We considered the benefits and critical aspects of remote working as two complementary aspects of the same construct, which is why in conducting the exploratory factor analysis we preferred an oblimin rotation that effectively established correlational links between the two latent dimensions. As for the extraction method, we used the Ordinary Least Squares (OLS) method to find the minimum residual solution. This leads to solutions very similar to the maximum likelihood, even for poorly behaved matrices. In addition, OLS has the advantage of recovering weak factors even in the presence of large sampling errors, by producing fewer borderline estimations (Briggs and MacCallum 2003; MacCallum 2001; MacCallum et al. 2007).

Thus, both eigenvalues and screeplot criteria recommended the two-factor solution: they showed SS loadings and eigenvalues > 1 and the screeplot indicated a very large difference in explained variance between the second and third dimensions. More specifically, the only two eigenvalues greater than the value of one belonged to the first two dimensions (3.89 and 2.90) and they cumulatively explained the 52.4% of the variance (Table 6).

**Table 6:** Factor loadings of EFA

Item	Dimension 1 (Benefits)	Dimension 2 (Disadvantages)
Item 1	**0.67**	− 0.08
Item 2	**0.49**	− 0.12
Item 3	**0.77**	− 0.03
Item 4	**0.80**	− 0.00
Item 5	**0.71**	0.14
Item 6	**0.81**	− 0.00
Item 7	**0.53**	0.02
Item 8	− 0.13	**0.65**
Item 9	0.03	**0.73**
Item 10	− 0.00	**0.74**
Item 11	− 0.01	**0.77**
Item 12	0.03	**0.83**
Item 13	0.16	**0.74**
Item 14	− 0.17	**0.70**

In bold the strongest correlations between item and latent dimensions

Dimension 1 explained 27.7% of the total variance, while dimension 2 explained 24.4%. The strongest factor loadings of items 1-7 ranged from 0.49 to 0.81, and those of item 8-14 ranged from 0.65 to 0.83. Reliability analysis revealed that the two subdimensions had an excellent internal consistency index, with Cronbach’s $\alpha$ and McDonald’s $\omega$ = 0.86 for the Benefits subscale and Cronbach’s $\alpha$ and McDonald’s $\omega$ = 0.90 for the Disadvantages subscale. Thus, we decided to consider two-dimensionality as the main structure of our scale.

## Study 2: confirmatory factor analysis

In line with the EFA suggestions, a Confirmatory Model was tested on a sample of 677 individuals. Again, the sampling was snowballing procedure using a non-probabilistic method. The subjects who completed the online questionnaire were all Italian workers who had worked from home during the period of the health emergency. Finally, the measurements were performed in the period between November 2020 and April 2021. In this case, too, sampling was done by snowball procedure using a non-probabilistic method. The subjects who completed the online questionnaire were all Italian workers who had experience of working from home during the health emergency period. Finally, the measurements were carried out in the time period between November 2020 and April 2021.

The percentage of men was 45.8% and the percentage of women was 54.2%. Most of the respondents had a university degree (50.8%) and a high school diploma (33.5%). 63.8% of the subjects were in a relationship, while 36.2% affirmed the opposite. 58.5% did not have dependent child. 62% had a permanent employment contract, 21.4% had a temporary contract, and 8.1% were self-employed. Regarding work, 57.9% of respondents worked in a private company and 42.1% in a public organization. The majority of the sample (34.7%) reported their workplace was between 1 and 5 kilometers away from their residence and 30.7% between 10 and 25 kilometers. The average age was 39.5 years, with a SD = 12.3 and an age range between 18 and 68 years.

Asimmetry and kurtosis ranged within ± 1.96 for all items, as represented in Table 7. Because the number of rows with missing data was insignificant compared with the total number of observations (44 subjects with missing data, about 6.5%), it was decided to eliminate these data from the final data set of 633 subjects so as not to replace them with other indices.

**Table 7:** Principal descriptive statistics of the CFA sample

	Mean	Std. error mean	SD	Skewness	Std. error skewness	Kurtosis	Std. error kurtosis
BEN1	3.03	0.03	0.76	− 0.40	0.09	− 0.33	0.19
BEN2	3.41	0.03	0.68	− 0.98	0.09	0.74	0.19
BEN3	2.76	0.03	0.90	− 0.21	0.09	− 0.76	0.19
BEN4	3.10	0.03	0.73	− 0.34	0.09	− 0.50	0.19
BEN5	2.27	0.04	0.94	0.31	0.09	− 0.77	0.19
BEN6	2.46	0.04	0.94	0.09	0.10	− 0.87	0.19
BEN7	2.97	0.03	0.85	− 0.46	0.10	− 0.46	0.19
CRIT1	2.93	0.03	0.83	− 0.31	0.09	− 0.61	0.19
CRIT2	2.55	0.04	0.93	− 0.01	0.10	− 0.86	0.19
CRIT3	2.68	0.03	0.90	− 0.21	0.09	− 0.71	0.19
CRIT4	2.28	0.04	0.93	0.23	0.09	− 0.79	0.19
CRIT5	2.38	0.03	0.88	0.20	0.09	− 0.66	0.19
CRIT6	1.97	0.04	0.92	0.63	0.09	− 0.49	0.19
CRIT7	2.51	0.04	0.93	− 0.03	0.09	− 0.84	0.19

**Fig. 1:**
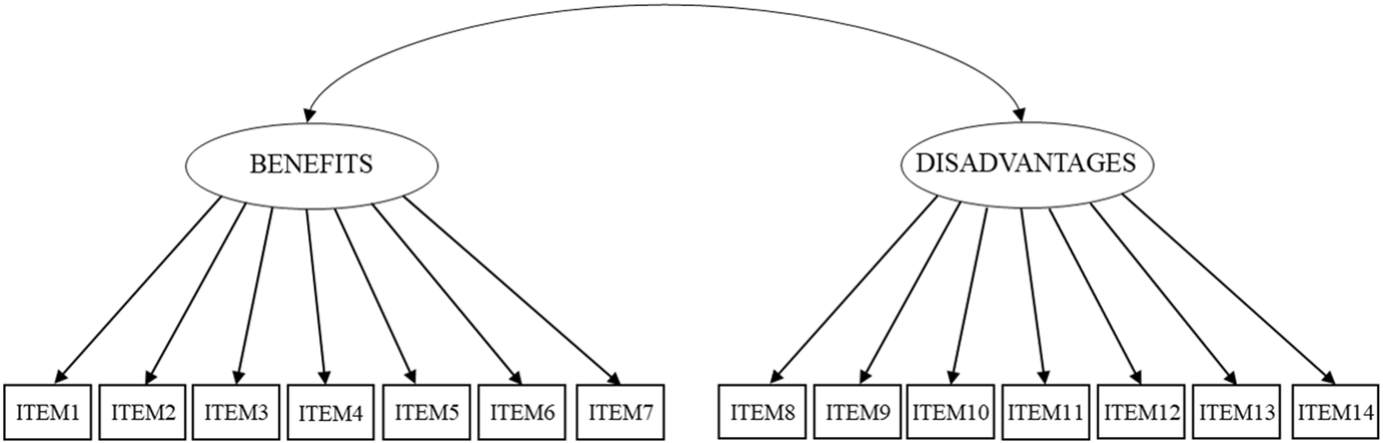
Confirmatory factor model

Since the skewness and kurtosis indices suggested a substantial normality of the data, we performed the CFA using the ML estimator and including two dimensions (Benefits and Disadvantages of remote working, 7 items each), as shown in Fig. 1.

### Results

Regarding the assessment of model quality, Lt and Bentler (1998) recommend relying on fit indices that have different measurement properties: therefore, in addition to the $\chi ^{2}$ statistic, the model evaluation was based on the Comparative Fit Index (CFI), the Tucker-Lewis Index (TLI), the residuals-based fit index SRMR and the RMSEA, i.e., the indices that also perform best according to the recent study by Jackson et al. (2009). Table 8 shows the fit indices for the tested models. $Model_1$ is a one-factor structure, i.e., a model in which benefits and disadvantages are considered as a single dimension rather than two separate factors, $Model_2$ considers benefits and disadvantages as two different dimensions of the remote working construct (two-factor structure with no residual correlations) and $Model_3$ is a two-factor structure with residual correlations. Given the poor fit indices of the model with one solution as well as the two-factor solution without residual correlations, and in line with the two-factor structure previously suggested by EFA, $Model_3$ was chosen over the alternatives.

**Table 8:** Alternative models

Model	$\chi ^{2}$	df	p	CFI	TLI	SRMR	RMSEA	Comparison	$\Delta \chi ^{2}$	$\Delta$ p
$Model_1$	2197	77	.000	0.42	0.31	0.22	0.21			
$Model_2$	442	76	.000	0.90	0.90	0.06	0.09	$M_3 - M_1$	1775	< .000
$Model_3$	335	73	.000	0.93	0.93	0.06	0.08	$M_3 - M_2$	107	< .000

**Fig. 2:**
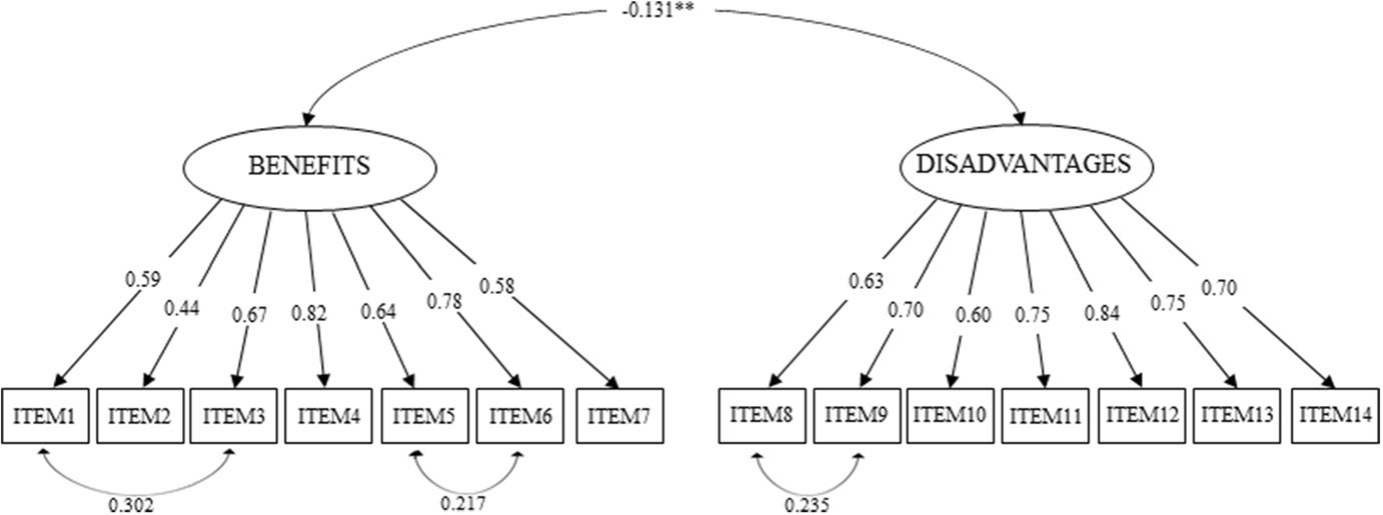
Factor loadings, factor correlation and residual covariances of the final Benefits-Disadvantages model. All parameters except factor correlations ($$^{**} p < 0.01$$) were significant at $$p < 0.001$$

In terms of structural model, we found good fit indices in $Model_3$, with $\chi ^{2}$ = 335 (.000), CFI = 0.93, TLI = 0.93, SRMR = 0.06, RMSEA = 0.08 (CI = 0.07; 0.09). Thus, $Model_3$ performed better with 3 residual correlations to be added: one between Item 1 and 3, that is, both items concerning one’s private life (Benefits, $Std.Est_{Ben_1 \leftrightarrow Ben_3}$ = 0.30[0.22; 0.39]), between Items 5 and 6 (Benefits, $Std.Est_{Ben_5 \leftrightarrow Ben_6}$ = 0.27[0.09; 0.34]), and a further residual correlation between Items 8 and 9, both concerning reduced visibility and isolation at work (Disadvantages, $Std.Est_{Dis_5 \leftrightarrow Dis_6}$ $= 0.23 [0.15;0.32$]). Item 1 of the benefits, in fact, concerns better conciliation while item 3 concerns stress. As widely noted in the literature, in fact, a healthier home-work balance is closely associated with a decrease in stress (Westman et al. 2004). Similarly, several studies and systematic reviews, such as those of Sousa-Poza and Sousa-Poza (2000) and Faragher et al. (2013), confirm that good relationships with colleagues and superiors are positively associated with job satisfaction, which is why we hypothesised a residual correlation between items 5 and 6. Finally, items 8 and 9, both belonging to the disadvantages dimension, were correlated in the residuals since, as Mulki et al. (2009) and Barsness et al. (2005) state, one of the most critical elements of remote working is the sense of isolation, both social and physical, which is often associated with perceived reduced opportunities and feeling of decreased psychological meaning ascribed to one’s work. All the estimates resulted to be statistically significant and are reported in Fig. 2.

Loadings ranged from 0.44 to 0.82 for the *Benefits* dimension and from 0.60 to 0.84 for the *Disadvantages* dimension. Bootstrap validation was performed through 5000 resamplings and we found that all saturations were significant, as shown in Table 9. Reliability analysis confirmed the goodness of internal consistency of the data: the *benefits* items reached Cronbach’s $\alpha$ = 0.83 and McDonald’s $\omega$ = 0.84, while the *disadvantages* items achieved Cronbach’s $\alpha$ and McDonald’s $\omega$ = 0.88. Finally, with regard to composite reliability, we found that $CR_{BENEFITS} = 0.87$ and $CR_{DISADVANTAGES} = 0.88$.

**Table 9:** Bootstrapping validation, *n* = 5000

Item	Dimension	Loading	z value	p.value	Lower CI	Upper CI
Item 1	Benefits	0.59	18.18	0.000	0.53	0.66
Item 2	Benefits	0.44	10.10	0.000	0.35	0.52
Item 3	Benefits	0.67	23.41	0.000	0.61	0.72
Item 4	Benefits	0.82	37.18	0.000	0.77	0.86
Item 5	Benefits	0.63	19.46	0.000	0.57	0.70
Item 6	Benefits	0.78	30.65	0.000	0.73	0.83
Item 7	Benefits	0.58	16.54	0.000	0.51	0.64
Item 8	Disadvantages	0.63	23.61	0.000	0.57	0.68
Item 9	Disadvantages	0.70	28.32	0.000	0.65	0.75
Item 10	Disadvantages	0.60	20.63	0.000	0.54	0.66
Item 11	Disadvantages	0.75	30.62	0.000	0.70	0.80
Item 12	Disadvantages	0.84	49.84	0.000	0.81	0.87
Item 13	Disadvantages	0.75	31.23	0.000	0.70	0.79
Item 14	Disadvantages	0.70	26.31	0.000	0.65	0.75

#### Discriminant and convergent validity

We therefore tested *discriminant* validity through correlations between manifest indicators and latent variables, as suggested by Gefen and Straub (2005). Moreover, the latent constructs are explained to more than 50% of the variance by the manifest indicators, as $AVE_{BENEFITS} = 51.0\%$ and $AVE_{DISADVANTAGES} = 52.4\%$, which is greater than the Maximum Shared Variance between latent variables (0.02).

**Table 10:** Cross-loadings of CFA sample

Item	Dimension	Benefits	Disadvantages
Item 1	Benefits	**0.64**	− 0.14
Item 2	Benefits	**0.48**	− 0.11
Item 3	Benefits	**0.72**	− 0.08
Item 4	Benefits	**0.88**	− 0.13
Item 5	Benefits	**0.68**	− 0.01
Item 6	Benefits	**0.84**	− 0.10
Item 7	Benefits	**0.62**	− 0.06
Item 8	Disadvantages	− 0.19	**0.67**
Item 9	Disadvantages	− 0.05	**0.74**
Item 10	Disadvantages	− 0.09	**0.64**
Item 11	Disadvantages	− 0.16	**0.80**
Item 12	Disadvantages	− 0.09	**0.89**
Item 13	Disadvantages	0.01	**0.79**
Item 14	Disadvantages	− 0.22	**0.75**

Discriminant validity was confirmed since the strongest correlations (in bold) were between item and the relative dimension

Table 10 shows the correlations between the manifest variables, or the items of the scale, and the two latent dimensions. As the bold values show, the items concerning the benefits of remote working correlate most strongly with the dimension to which they belong, as do the items of the disadvantages.

Finally, to test convergent validity, correlations between the new measure proposed in this study and other constructs considered related in the relevant literature were analysed. Although there are currently no scales that summarise the advantages and disadvantages of remote working, it is known that its positive effects include autonomy, better job and life satisfaction. On the other hand, stress due to new technologies, emotional exhaustion and increased workload are disadvantages. Job autonomy was measured with 4 items by Breaugh (1985), technostress trough 11 items by Molino et al. (2020), emotional exhaustion using 5 items by Sirigatti et al. (1988), workload with 3 items by Melin et al. (2014), and life satisfaction and job satisfaction with one ad hoc item each. Indeed, the empirical evidence of the results obtained by the correlation matrix in Table 11 shows that the benefits of remote working are positively and significantly related to job autonomy (*r* = 0.20, p < 0.000), life satisfaction (*r* = 0.58, p < 0.000) and job satisfaction (*r* = 0.52, p < 0.000). In contrast, high levels of remote working benefits correspond to low levels of technostress (*r* = − 0.32, p < 0.000), emotional exhaustion (*r* = − 0.35, p < 0.000), and workload (*r* = − 0.23, p < 0.000). The opposite trend is seen in the disadvantages of remote working, which increase with low autonomy (*r* = − 0.35, p < 0.000), life satisfaction (*r* = − 0.30, p < 0.000), and job satisfaction (*r* = − 0.32, p < 0.000), and are positively correlated with technostress (*r* = 0.56, p < 0.000), emotional exhaustion (*r* = 0.31, p < 0.000), and workload (*r* = 0.10, p < 0.05). These results, therefore, indicate that the scale has the potential to identify the hypothesised latent constructs and demonstrate statistical correlations that are consistent with the literature on this topic.

**Table 11:** Correlations of latent variables.

	Autonomy	Technostress	Emotional Exhaustion	Workload	Life satisfaction	Job satisfaction
Benefits	0.20	− 0.32	− 0.35	− 0.23	0.58	0.52
Disadvantages	− 0.35	0.56	0.31	0.10$$^{**}$$	− 0.30	− 0.32

All correlations were significant at p $$< 0.001$$ except for the one labelled with $$^{**}$$ (significant at p $$< 0.05$$)

In order to test whether the items had the same meaning for different groups, we tested measurement invariance (Lecciso et al. 2019) across *Gender* ($N_{F}$ = 367 and $N_{M}$ = 310), and *sector* ($N_{PUBLIC}$ = 285 and $N_{PRIVATE}$ = 392), aiming at ruling out the possibility that aspects of remote working (e.g., work-family balance, work recognition) might be perceived differently by gender or sector typology. According to the recent review by Putnick and Bornstein (2016), the difference in fit indices is reported in Table 12.

**Table 12:** Measurement Invariance: Differences in fit indices

Differences	df	$\Delta$ CFI	$\Delta$ TLI	$\Delta$ AIC	$\Delta$ BIC	$\Delta$ RMSEA	$\Delta$ SRMR
*Gender*							
metric - configural	12	− 0.001	0.006	− 10.048	− 63.107	− 0.003	0.003
scalar - metric	12	− 0.005	0.001	4.725	− 48.335	0.000	0.002
strict - scalar	14	− 0.001	0.006	− 11.465	− 73.368	− 0.003	0.001
*Sector*							
metric - configural	12	− 0.004	0.003	4.257	− 48.802	− 0.001	0.008
scalar - metric	12	− 0.013	− 0.007	37.51	− 15.55	0.003	0.003
strict - scalar	14	− 0.002	0.006	− 6.389	− 68.292	− 0.002	0.000

In measurement invariance testing, we did not consider the difference between models in $\chi ^2$, as it is very sensitive to sample size (Brannick 1995; Kelloway 1995). According to Cheung and Rensvold (2002), Chen (2007) and Chen et al. (2008), we preferred $\Delta$ CFI, $\Delta$ RMSEA and $\Delta$ SRMR, which should not exceed the difference of 0.01, 0.015 and 0.030, respectively. Table 12 shows that the differences between configural, metric, scalar and residual models fall within the proposed range, except for scalar and metric models in sector invariance in only $\Delta$ CFI. Thus, we considered measurement invariance to be respected.

## Discussion and conclusions

The results of the analysis show that the hypothesised scale can be considered a good psychometrical questionnaire to detect the benefits and disadvantages that remote working can bring to workers and very often has brought in times of pandemic.

The main goal of the study was to provide researchers and practitioners with a scale with appropriate psychometric requirements for widespread use. To meet this challenge, we initiated a process of instrument development that resulted from the realisation that there are scales that measure the psychological aspects of remote working using a constellation of questionnaires rather than a single, streamlined instrument, as we found in our review of the literature on the topic. Indeed, to our knowledge, there is currently no scale that summarises the main advantages and disadvantages of remote working in a single indicator. The first part of the development of the questionnaire therefore aimed to focus on the existing literature, which experienced an exponential increase during the Covid-19 pandemic, in order to identify the core constructs that are commonly and empirically associated with remote working. A subsequent focus group phase with privileged witnesses allowed us to determine whether the identified constructs might encompass the actual needs of workers. Subsequent exploratory analyses, resulting in two studies with a total of 605 workers, paved the way for amendments and integrations to the original pool of developed items in an ongoing monitoring process of scale development.

The final version of the instrument showed how the individual items of the questionnaire could be effectively classified into the two dimensions theorised in the literature, namely benefits and disadvantages. Finally, confirmatory analysis conducted on a sample of 677 workers showed that the main psychometric characteristics of the instrument were met in terms of factorial validity, reliability, convergent and discriminant validity, and invariance.

Therefore, at different stages of evaluation, the scale could provide useful insights into the complexity of the phenomenon under study, especially considering that it is invariant in terms of gender and sector. Regarding this aspect, the results of our analyses suggest that the questionnaire actually measures the proposed concepts, regardless of workers’ gender or sector, whether public or private. Finally, the study made it possible to establish how certain psychological constructs inherent to work can serve as key elements for the study of remote working. In this sense, it was possible to assess how autonomy is actually a protective element against negative outcomes (Signore et al. 2020), and increases the benefits perceived by workers. On the contrary, workload was found to be negatively associated with benefits (Ingusci et al. 2021) and positively associated with critical aspects (Molino et al. 2019), as well as with stress due to new technologies and emotional exhaustion Molino et al. (2019, 2020); Signore et al. (2021); Bondanini et al. (2020); Leonardi et al. (2010). These considerations serve as a double confirmation: from the point of view of convergent validity, since some items include aspects such as autonomy and stress due to new technologies, and from a practical point of view, since these constructs are known and have been empirically studied in relation to remote working.

Although the relationship between remote working and positive or negative outcomes is still unclear today, as much depends on the conditions of the workers and the companies in which these policies are embedded, the pandemic context has created a very similar situation worldwide, providing food for thought that cannot fail to be taken into account. In this regard, our scale, which has met expectations since it is based on a bifactorial structure (divided into benefits and disadvantages), could easily be used to study the consequences of remote working in more detail and to identify any risk situations at an early stage. Although the fight against the pandemic seems to have reached a turning point, many companies, that have directly experienced the benefits of remote working, especially in terms of economic savings, are thinking of adopting this work method permanently. In this sense, however, it is necessary to reflect on the fact that the tool we intended to validate and the existing literature on the subject allow us to reiterate that the benefits and disadvantages are sides of the same coin inserted in the management of remote working itself, so it is necessary to be sparing with any reflection on the subject.

## Limitation and practical implications

The generalisability of the results is undermined by some critical issues, as convenience sampling, heterogeneity of the samples, self-report measures and the use of parametrical methods of analysis. More specifically, participants were enrolled in the study by means of a non-probabilistic sampling with snowball nature, characterised by the fact that it was non-random and included mainly Italian workers, although from different geographical areas. Additionally, the professionals included in the study are very diversified. Among the various participants, there are self-employed workers who, due to the nature of their profession, benefit from a certain degree of autonomy and flexibility. Thus, future studies need to confirm the validation of the questionnaire by considering these aspects.

Moreover, answers to the questions on the psychological constructs analysed, as well as to the questionnaire itself, are self-reported, and therefore subject to common method-bias (Jordan and Troth 2020). Further studies could use objective indices, such as measuring supervisor satisfaction or employees’ absenteeism. Although in the present study it was possible to investigate the psychometric fit of the scale by means of robust parametric methods, the factorial structure of the questionnaire and relationships with other constructs could be investigated using other approaches and statistical techniques, such as high-order constructs (Ciavolino and Nitti 2013) or composite indicators (Carpita et al. 2020), depending on the characteristics of future research designs.

Another limitation of the present study concerns the culture in which the questionnaire was validated. Since this study was limited to Italian workers, the scale was administered in Italian and in the specific reference context. Therefore, in terms of cross-cultural validation of the instrument, further studies could investigate invariance also as a function of language, together with gender and sector. Finally, the questionnaire did not include questions about the time period when remote working was first experienced (whether before or during the pandemic). This is an aspect that future studies could explore using measurement invariance to understand whether perceptions of benefits or critical aspects depend on the historical period in which remote working was first practiced.

Nevertheless, the consistent results of the scale in the different studies allowed the operationalisation of a tool that can be used to identify the benefits and disadvantages associated with remote working. For this reason, it can be a streamlined and psychometrically valid instrument that can be used to identify and take action at an early stage on the potential difficulties arising from remote working, while also identifying positive aspects that can be implemented to improve organisational well-being.

Based on the existing literature, the RW-B&D scale allows to investigate the positive and negative aspects related to remote working. The tool, therefore, assumes a crucial role, especially in a period still marked by the changes resulting from the recent pandemic. In view of a gradual return to normality and considering that several organisations have already considered the idea of resorting to remote working by virtue of the economic savings and psychological benefits for workers, the questionnaire may be an appropriate tool for researchers and practitioners to investigate the positive or negative impact of this form of work, and, if necessary, to monitor its effects and take timely action. To cope with the pandemic period, workers should need more job resources to balance job demands. Organizations could implement top-down (or bottom-up) interventions to provide workers with the support they need and to take care of their health: they should provide immediate resources, such as information about working from home, or information on assistance programs, counselling or training. They should also provide psychological support, by offering feedback on their work and regular contact (Kniffin et al. 2021).
